# Case report on energy drink consumption among Health Sciences University students in Gauteng Province, South Africa

**DOI:** 10.1186/s40985-020-00129-2

**Published:** 2020-05-13

**Authors:** Lucy Fernandes, Kebogile Mokwena, Busisiwe Ntuli

**Affiliations:** Department of Public Health, Sefako Makgatho Health Sciences University, P.O. Box 215, Medunsa 0204, Pretoria, South Africa

**Keywords:** Energy drink, Energy drink mixed with alcohol, Students

## Abstract

**Background:**

There are claims that energy drink (ED) consumption can bring about an improvement in mental functioning in the form of increased alertness and enhanced mental and physical energy. These claims address the lifestyle of a student of sleep deprivation and academic pressure with ED consumption becoming a popular practice amongst students. The study’s objectives were to determine the prevalence, reasons for, and patterns of ED and alcohol mixed with ED (AmED) consumption amongst university students.

**Case presentation:**

Registered students of the Health Sciences University, Gauteng Province, South Africa, formed the study population where this quantitative cross-sectional survey conveniently collected data by means of previous validated self-administered questionnaires from 490 students representing the diverse demographics of the university. Questions on the socio-demographic profile of the participants; pattern of alcohol use; reasons, pattern, and preferred types of EDs use; and the pattern, reason, and experience of AmED use during the past 12 months were asked. Frequency and percentages of distributions were determined, and the possible factors contributing to ED consumption were computed using the chi-square test. Results indicated that a total of 58% of students were consuming EDs mainly to stay awake (31%), to be more alert (14%), and to help with concentration (15%), 69% were consuming alcohol, and 16% were consuming AmEDs.

**Conclusion:**

There is an urgent need for an appropriate public health prevention intervention so that ED consumers can make informed choices when indulging in these health behaviors before the seemingly harmless consumption of ED amongst students becomes a public health issue.

## Background

Globally, the consumption of energy drinks (EDs) has gained popularity since the 1997 debut of Red Bull© [1]. In South Africa, EDs also form a fast-growing segment of the soft drink market where figures are indicating that between 2009 and 2014, the annual volume of sports and EDs sold rose from 97.7 million liters to 167.7 million liters, a rise of approximately 2 to 3 liters per capita in only 5 years [2]. In South Africa, EDs are easily accessible and freely available in shops and is even sold on the crossroads by street vendors with the most common brands of EDs available being: Monster©, Play©, Red Bull©, Full©, Mother©, Wired©, Energade©, MoFaya©, and Reboost© [3].

The main ingredients of EDs (depending on the brand) include guarana, caffeine, ginseng, and small amounts of theophylline, tannins, taurine, and sugar [4]. It is important to note that EDs, where the key ingredient is caffeine, is not the same as sports drinks. By consuming EDs, the central nervous system is stimulated while sports drinks replace chemicals lost during sweating enabling a person to sustain physical activity for longer periods [5]. For the purpose of this study, an energy drink was defined as a “non-alcoholic beverage” marketed as boosting energy levels.

The consumption of the perceived, relatively harmless, EDs has become a public health concern [6–8]. The effects of caffeine are dose-related, with low to moderate doses of caffeine (20 to 200 mg) resulting in increased happiness, energy, alertness, and sociability [9] with no adverse health effects [10, 11]. While at higher doses (1000–1500 mg per day), caffeine is also a potent stimulant which could lead to the potential of caffeine intoxication and caffeine dependence [12]. Due to the variation in the amounts of caffeine in EDs and the inadequate product labeling, the likelihood of caffeine overdose is a reality which can be medically problematic [9]. Caffeine intoxication is characterized by headaches, sleep disorders, restlessness, tiredness and irritation, excitement, elevated blood pressure, incoherent and excited thoughts, and speech [1, 10, 12–15]. If caffeine intake is not properly monitored, it could lead to possible dependence accompanied by possible side effects and even harmful withdrawal symptoms [12, 13].

Global literature indicates that amongst students, ED consumption has become a very popular practice due to the marketing claims that EDs can bring about an improvement in mental functioning in the form of increased alertness, improved mood, and enhanced mental and physical energy [1, 14, 16, 17]. These claims fit in with the fast-paced lifestyle of a student of sleep deprivation and academic pressure [18]. Literature is now suggesting that between 34% and 80% of college students have used EDs in the previous month [1, 8, 19]. It is plausible that the excessive consumption of EDs is a marker for high-risk behaviors or may play a role in escalating risk behavior where EDs are used as mixers for alcohol (AmED), for example, Red Bull and vodka or Jägerbombs where a shot of Jägermeister is mixed with a ED [20].

AmED consumption has now become a relatively popular potential health risk behavior and emerging trend among young adults [14, 17, 21, 22]. The perceived benefits of AmED consumption are to quicken the onset of intoxication, to reduce fatigue after drinking, and to improve the taste of alcohol [17]. The negative consequences for AmED which may include alcohol dependence, binge drinking, the potential for sexually transmitted infections via high-risk sexual behavior, and substance use is well documented [8, 17, 23]. In part, the reason for AmED is because drinkers experience a different subjective response to the alcohol when drinking it as opposed to a beverage that is not mixed with caffeine and other stimulant ingredients [16].

Despite the potential negative health impacts of ED consumption and the dangers of AmEDs, research on caffeine in South Africa is very limited [24]. A setting, such as a university, is ideal due to the fact that students, of which some can be regarded as young adults, are the group specifically targeted by ED marketing [25, 26].

The aim of this study was to determine the prevalence and patterns of ED consumption amongst university students and determine the proportion of students partaking in AmEDs. Though this study was mainly about ED consumption, individuals with hazardous alcohol use patterns had to be identified in order to determine the prevalence of AmED. By gaining a deeper understanding of the prevalence and extend of ED consumption and the potential harmful practice of AmED amongst the students, this study will inform the development of health education interventions to increase the awareness of students about the potential harmful effects of ED consumption while addressing this emerging public health problem.

## Case presentation

This was a quantitative cross-sectional descriptive case study using a self-administered questionnaire. The setting was the Sefako Makgatho Health Sciences University (SMU) which is a comprehensive health sciences university. Participants were recruited from the five schools within the university, i.e., Medicine, Pharmacy, Health Care Sciences, Oral Health Sciences, and Science and Technology. The university is located in Pretoria, Gauteng Province, South Africa. SMU was established in 2015 and offers various Diploma, Bachelor, Masters, and PhD programs for undergraduate and postgraduate students.

Data were collected from a convenience sample of 334 undergraduate and 158 postgraduate students out of a total of 5812 registered students for 2018. The undergraduate students were recruited during their participation in an integrated course where all the schools in the university were represented, while the other students were registered for a postgraduate program in Public Health.

The research assistants who collected the data were trained on the study objectives, the ethics of recruitment, and data collection. At the end of the official lecture period, the background and aim of the study were explained by the research assistants and students were also informed that participation was anonymous and voluntary. After agreeing to participate, the students received a self-administered questionnaire which took about 10 min to complete. Part of the instructions on the question had a statement saying that “by completing this questionnaire, you are consenting to participate in the study.” The research assistants collected the questionnaires immediately after completion. The self-administered questionnaire was based on previously validated questionnaires [6, 10, 13] and the Alcohol Use Disorder Identification Test—Consumption (AUDIT-C) [27] (available in the public domain) all covering the topic of alcohol and/or ED consumption. The questionnaire had four sections.

1. The socio-demographic profiles of the participants: Data were captured by respondents providing their self-reported age and course that they were registered for. Limited options were provided to choose from for the level of study. Students younger than 25 years of age were regarded as “emerging adults” as per international age classification [28].
2. The pattern and type of ED consumed: The pattern was determined by asking how many times and the number of ED drinks per session the participant has consumed during the past 7 days. The options given were “every day,” “4–5 times a week,” “2–3 times a week,” “once a week,” and “depending on the need.” Options were given to choose from for the types of EDs consumed and the reasons for ED consumption. Descriptive statistics, i.e., frequency and percentages of distributions, were determined by using Epi InfoTM.
3. Alcohol use: For the question “How often did you have an alcoholic drink in the past year?” the responses were scored as follows: never (0 points), once a month (1 point), 2–4 times a month (2 points), 2–3 times a week (3 points), 4–5 times a week (4 points), and 6 or more times a week (4 points). For the question “How many alcoholic drinks did you have on a typical day when you were drinking in the past year?” the responses were scored as follows: 0 drinks (0 points), 1–2 drinks (0 points), 3–4 drinks (1 point), 5–6 drinks (2 points), 7–9 drinks (3 points), and 10 or more drinks (4 points). For the question “How often did you have 6 or more alcoholic drinks on one occasion in the past year?” the point allocated were as follows: never (0 points), less than monthly (1 point), monthly (2 points), weekly (3 points) daily, or almost daily (4 points). The scores for the three questions were added and students could receive a possible score ranging from 0 to 12. An overall total score of 5 or above was taken as an indication of hazardous or harmful drinking [27].
4. AmED: Participants were given options to choose from for the question on how many days did they have an ED and alcohol on the same occasion and/or in the same drink during the last year. Only those who mix alcohol and EDs were asked to choose between options pertaining to why they engage in this practice and how they felt after consuming AmEDs.

For inferential statistics, the relationship between the independent variable ED consumption, and other variables (gender, level of study, age, and use of alcohol) was computed using the odds ratio, 95% CI, and chi-square test with bivariate correlation with a *p* value < 0.05 considered to be significant.

The necessary institutional ethical clearance and approval was obtained from the Sefako Makgatho Health Sciences University Research and Ethics Committee reference number SMUREC/H/271:2017:IR. Participation was voluntary and anonymous after signing informed consent, and participants received no compensation.

## Results

### Demographic description of study participants

The majority of students were undergraduate (68%), females (71%), with 51% younger than 25 years of age, and in their final year of study (68%). Presented in Table 1 are the demographic characteristics of the participating students.

**Table 1:** Demographic information (*N* = 490)

		Frequency	Percentage
**Age**	19–24	251	51
	25–30	120	25
	31–36	43	9
	37–41	45	9
	43–49	20	4
	50–56	11	2
**Gender**	Female	346	71
	Male	144	29
**Study level**	Undergraduate	332	68
	Postgraduate	158	32
**Degree registered for**	Medicine (MBChB)	159	32
	Nursing Science (BCur)	34	7
	Pharmacy (B Pharm)	33	7
	Occupational Therapy	32	6
	Physiotherapy	29	6
	Human Nutrition and Dietetics	26	5
	Speech Language Pathology & Audiology	19	4
	Master of Public Health (MPH)	120	25
	Postgraduate Diploma in Public Health (PGDPH)	38	8
**Year of study**	1st year	104	21
	2nd year	55	11
	4th year	173	36
	6th year	158	32

### Prevalence and pattern of ED use

The ED consumption patterns are presented in Table 2 with more adults than emerging students consuming EDs (63% vs 53%), and the main brands consumed amongst the total population being Red Bull (29%) followed by Dragon (28%) which were mainly consumed depending on the need (72%).

**Table 2:** ED consumption patterns

	Emerging adult (***n*** = 251)		Adult (***n*** = 239)		Total population (***N*** = 490)	
	Frequency	%	Frequency	%	Frequency	%
**Consume energy drinks**						
Yes	134	53	151	63	285	58
No	117	47	88	37	205	42
**Number per typical sitting**						
One	118	88	137	91	255	90
Two	14	10	13	9	27	9
Three or more	2	2	1		3	1
**Number of times consumed in the past 7 days**						
Every day	2	2	3	2	5	2
4–5 times a week	15	11	20	13	35	12
2–3 times a week	10	7	11	7	21	7
Once a week	6	5	13	9	19	7
Depends on the need	101	75	104	69	205	72
Brands of ED consumed*						
Red Bull (80 mg caffeine/can)	39	29	44	29	83	29
Dragon (180 mg caffeine/can)	39	29	40	26	79	28
Reboost Power Play (80 mg caffeine /can)	32	24	35	23	67	24
Monster (80 mg caffeine/can)	22	16	34	23	56	20
Vitamin Water (2.5 mg/100 ml)	14	10	22	15	36	13

*Some students were consuming up to seven different types of EDs

### Reasons for ED use

Participants were given a list of possible reasons to choose from and the self-reported reasons for ED use are presented in Table 3 where 28% of adult students consumed it to be more alert versus the 2% amongst emerging adults. Adult students were also hoping that they will perform better in the exam (8% vs 3% amongst emerging adults).

**Table 3:** Participants self-reported reasons for ED use

	Emerging adult (***n*** = 251)		Adult (***n*** = 239)		Total population (***N*** = 490)	
Reason for ED consumption^**#**^	Frequency	%	Frequency	%	Frequency	%
To stay awake	77	31	76	32	153	31
Helps to concentrate during study	37	15	37	15	74	15
To be more alert	5	2	66	28	71	14
To compensate for insufficient sleep	22	9	34	14	56	11
It gives long-lasting energy all day	24	10	31	13	55	11
Like the taste	22	9	30	13	52	11
It quenches thirst	17	7	21	9	38	8
It makes the user feel refreshed	18	7	16	7	34	7
Helps with better performance in the exam	8	3	19	8	27	6
It gives an instant energy rush	14	6	9	4	23	5
It replenishes electrolytes after exercise	8	3	14	6	22	4
Curiosity	8	3	8	3	16	3
To enable a person to enjoy an all-night party	4	2	8	3	12	2
Makes user feel healthy	5	2	4	2	9	2
To feel better after a hangover	3	1	4	2	7	1

^#^Students could respond to all reasons applicable to them

### Prevalence and pattern of alcohol and AmED consumption

Presented in Table 4 are the results for the patterns of ED consumption, alcohol consumption, and AmED consumption with 76% of participants who were drinking EDs also drinking alcohol without mixing, 16% were mixing the two, and 7% of participants consuming alcohol were exposed to hazardous or harmful drinking.

**Table 4:** Patterns of ED, alcohol, and AmED consumption

	Emerging adult (***n*** = 251)		Adult (***n*** = 239)		Total population (***N*** = 490)	
Consumption	Frequency	%	Frequency	%	Frequency	%
Drinking EDs	134	53	152	64	286/490	58
Drinking alcohol	161	64	179	75	340/490	69
Drinking ED and alcohol but no mixing	97/134	72	121/152	79	218/286	76
AmEDs	25/134	19	21/152	14	46/286	16
Hazardous/harmful alcohol consumption^$^	12/161	7	12/179	7	24/340	7
Hazardous/harmful alcohol consumption$ + EDs	12/12	100	10/12	83	22/24	92
No ED and no alcohol	79/251	31	50/239	21	129/490	26

^$^According to Alcohol Use Disorder Identification Test—Consumption (AUDIT-C)

### Reasons, feelings, and risky behavior after consuming AmED

Presented in Table 5 are the reasons for consuming AmED, the experiences after the consumption of AmED, and the reported risky behaviors after the consumption of AmEDs.

**Table 5:** Reasons, feelings, and risky behavior after consuming AmED (*N* = 50)

	Frequency	Percentage
**Reasons for mixing EDs with alcohol**		
To stay awake	13	26
User feel less tired	10	20
To hide the flavour of alcohol	10	20
User like the taste of AmED	10	20
User can drink more and don’t feel so drunk	8	16
User need more energy in general	7	14
The mix can treat or cure a hangover	5	10
User enjoy the effects of the mix	4	8
Other reason/s	3	6
**Feeling after consuming AmEDs**		
Was full of energy	16	32
There was no difference	7	14
Had trouble sleeping/insomnia	5	10
Had stomach pain/stomach irritation	2	4
Heartbeat was irregular/racing heart	2	4
Had to seek medical help	2	4
Other	4	4
**Unprotected sex after consuming AmED**		
No	16	32
Yes	2	4
Can’t remember	3	6
**Use drugs for recreational purposes**		
No	46	92
Yes	4	8

The main reason for AmED was to stay awake (26%) and to feel less tired (20%) with 32% feeling full of energy after consumption. The minority (4%) were involved in unprotected sex or could not remember the incident, while 8% were using illicit drugs for recreational purposes.

### Possible factors related to ED consumption

The results of the chi-square analysis are presented in Table 6 indicating that alcohol use and age were some of the possible factors related to ED consumption.

**Table 6:** Possible factors related to ED consumption

Variable	Odds ratio	***p*** value	95% CI
Gender	1.0433	0.8329	0.7037–1.5468
Use alcohol	21.7048	**< 0.001**	12.8054–36.7889
Undergraduate or postgraduate	1.4080	0.8518	0.9530–2.0802
Emerging adult vs adult	1.5255	**0.0219**	1.0623–2.1906

## Discussion 

The university environment is a distinct setting for the use of EDs due to market claims of increased alertness and enhanced mental and physical energy [14]. These claims of temporary benefits hold the promise of assisting students to study which could lead to better academic performance [25]. Research within college populations suggests that between 34% and 58% of college students use energy drinks [1, 8, 25]. Amongst the participants of this study, 58% reported the consumption of ED during the last week which was in agreement with the prevalence rates reported by similar studies [9, 14, 25]. The main reasons for consuming EDs were to stay awake (31%), to be more alert (14%), and to help with concentration when studying (15%) which is similar to other studies [29] where 70% of students were consuming EDs for vitality and to be more alert (21%) [11]. A significant difference in ED consumption (*p* = 0.0219) was found between emerging adults, the group specifically targeted by marketing agencies [25, 26] and adult students.

Of those who were drinking EDs, 10% indicated that they were consuming more than one ED per sitting. Although this figure is much less than the 51% college students surveyed in another study [1], the problem is that the caffeine content of a single ED can range from 80 to 180 mg per can or bottle implying that more than one ED per day (depending on the brand) could potentially pose a health hazard to the consumer due to caffeine overdose [29]. Chronic toxicity due to prolonged exposure at levels greater than 500–600 mg a day [26] can have harmful detrimental physical consequences to the consumer as described before.

Although the main aim of this study was to determine ED consumption, the results of this study indicate that 69% of the students were consuming alcohol. The only factors related to ED consumption were alcohol consumption (*p* ≤ 0.001) and being younger than 25 years of age (*p* = 0.0219), revealing that those who consume EDs were 21.70 times more likely to also consume alcohol. These results are consistent with previous research supporting a link between caffeine consumption and later alcohol use among adolescents and college students [9, 14, 16, 23, 25]. But as stated in the literature, the specific nature of the relationship between ED consumption and alcohol consumption cannot be established as the correlations between ED and alcohol consumption do not imply that the one causes the other [9, 16, 30]. Though the thought is that when a person (especially a young person) consumes one beverage that changes how they feel (caffeine) they may be particularly likely to use another beverage that changes how they feel (alcohol) [30]. Due to the fact that only a small number of factors were examined, there is a possibility that other factors that were not tested for could be correlated to ED consumption.

When compared to published literature where it is estimated that between 24% and 51% [1, 17, 25] of college students reported consuming AmED during the past month, the current study finding of 16% of AmED use is relatively low. This corresponds with a review article [31] aimed to put into perspective the current scientific evidence on the combined use of AmEDs where it was concluded that a relative minority of students take part in the practice of AmEDs. The functional reasons given by the students for the combined use were to feel less tired, to stay awake, and to hide the taste of alcohol, while the motivation to get drunk faster and be able to drink more also corresponds with the literature [8]. However, consuming AmEDs is potentially very dangerous as AmED typically contain greater quantities of caffeine, which increases the user’s risk of experiencing negative side effects [25]. It is suggested that ED offset the drowsiness associated with alcohol consumption resulting in the user feeling less intoxicated which could lead to the possibility that the user may underestimate their level of intoxication and related impairment [11, 22]. For the user, there is an elevated risk of alcohol-related injury such as blacking out and alcohol poisoning [22] and the possibility of alcohol dependence as the consumption of AmED beverages has been shown to increase the desire for more alcohol [17]. Consuming AmED is positively associated with binge drinking, and binge drinking increases the risk of acute and chronic alcohol-related problems including risky sexual behaviors [8, 14, 22, 23]. Of the 16% of students that were consuming AmED, 92% were classified as partaking in hazardous or harmful drinking confirming the association between ED usage and high-risk drinking [9, 11].

Furthermore, according to the literature, those who consume AmEDs tend to have a greater involvement in drug use and have higher levels of sensation-seeking, compared with students who do not consume EDs [15]. Eight percent of the participants in the current study who were consuming AmEDs openly admitted that they were using drugs for recreational purposes and 10% who were consuming AmED engaged in unprotected sex or could not remember if they have used protection during the sexual encounter. Given the high HIV infection rates in sub-Saharan Africa where it is estimated that 19.0% of adults aged 15–49 is HIV positive [32], unprotected sex has public health implications for the spread of sexually transmitted diseases, including HIV, and unplanned pregnancies [8, 21].

### Implications

If the perceived harmless consumption of ED is indeed linked to a greater involvement in alcohol and drug use, then the public health consequences of unregulated EDs that are freely available, might be severe, and pose an important threat to the health of students.

### Limitations

Due to the convenience sampling of students from one health sciences university where their consumption practices could be different than students enrolled in programs not related to health, and the fact that 71% of the participants were female, generalizations beyond this sample may not be warranted. Despite anonymous participation, the self-report data used in this study may be subjected to social desirability bias as participants may have over or under reported the high-risk sexual behavior and drug use. Due to poor or incomplete memory recall, recall bias could be a factor. Due to the use of cross-sectional data, causal relationships could not be determined. To establish risky behavior, questions related to drug use and unprotected sex were only asked from students who were mixing alcohol and EDs.

## Conclusion and recommendations

In this study, 58% of the students were consuming EDs mainly to stay awake (31%), to be more alert (14%), and to help with concentration when studying (15%). Sixty-nine percent of students were consuming alcohol and 16% were consuming AmEDs.

It is of the utmost importance that students have to be informed about the possible serious negative consequences that are associated with the regular consumption of EDs; warning them about the side-effects and symptoms of caffeine withdrawal and the potential harm they are exposed to when consuming AmEDs which is becoming an increasing popular behavior.

## Data Availability

Please contact corresponding author for data requests.

## Abbreviations

AmEDs: Alcohol mixed with energy drinks
AUDIT-C: Alcohol Use Disorder Identification Test—Consumption
B Cur: Bachelor of Nursing Science
B Pharm: Bachelor of Pharmacy
EDs: Energy drinks
MBChB: Bachelor of Medicine and Bachelor of Surgery
MPH: Master of Public Health
PGDPH: Postgraduate Diploma in Public Health
SAMRC: South African Medical Research Council
